# Safety monitoring method of moving target in underground coal mine based on computer vision processing

**DOI:** 10.1038/s41598-022-22564-8

**Published:** 2022-10-25

**Authors:** Pengfei Xu, Zhiqing Zhou, Zexun Geng

**Affiliations:** 1 Department of Information Engineering, Pingdingshan University, Pingdingshan, 467000 Henan China; 2 Faculty of Engineering, Built Environment & Information Technology, SEGI University, Kuala Lumpur, Malaysia

**Keywords:** Environmental sciences, Environmental social sciences

## Abstract

Coal is one of the main energy sources in China. The country attaches great importance to the development of coal mining industry, and coal production is on the rise. At the same time, mine safety accidents are becoming more and more frequent, and the country is paying more and more attention to mine safety accidents. The underground environment of coal mine is complex, noisy and uneven, and there will be problems such as occlusion and high false detection rate during video monitoring. In order to ensure the safety of underground personnel, moving target detection and tracking based on video monitoring information is of great significance for coal mine safety production. The purpose of this paper is to study how to analyze and study the monitoring of moving targets in coal mines based on computer vision processing, and describe the image processing methods. This paper puts forward the problem of target monitoring, which is based on image processing, and then elaborates on the concept of image enhancement and related algorithms. From the average gradient, the algorithm in this paper is 56.60% higher than the histogram equalization algorithm, and 68.26% higher than the dark primary color prior dehazing algorithm. and designs and analyzes cases of image enhancement in coal mines. The experimental results show that the information entropy of the algorithm in this paper is 31.10% higher than that of the dark primary color prior dehazing algorithm, and 18.72% higher than that of the histogram equalization algorithm. It can be seen that the algorithm in this paper can achieve better enhancement effect.

## Introduction

Due to the special underground environment of the coal mine, the video information is different from the general ground scene. These problems are due to the darkness of the coal mine, resulting in low illumination of the video information, even with all-weather artificial lighting 21. However, different from natural light imaging, the illumination is obviously insufficient: second, due to the influence of factors such as mine dust and humidity, the underground video information is not clear, and the brightness near the light is relatively large, so the video imaging will appear white, and the video in other places without light irradiation is very dark, resulting in the blurred outline of the target and difficult to identify. Because the monitoring camera under the mine is relatively simple, and the video information collected is basically gray and black, which brings many difficulties to the detection algorithm that needs to extract features by color. Moreover, the underground monitoring camera is usually fixed, so when the target position changes, it will be affected by occlusion. Moreover, because the color of the target and the background are too close, when the posture of the target changes, It is difficult to apply the commonly used human contour recognition and modeling. In the gray environment under the mine, the current detection algorithm is likely to only detect bright areas, which will bring a lot In recent years, video image processing technology has developed rapidly. It is increasingly important to integrate video processing technology into mine monitoring to make mine monitoring more intelligent. The monitoring equipment has built-in video image processing technology, which can reduce the workload of personnel and improve the working efficiency of the monitoring equipment, thereby better guaranteeing the safe production of coal. This paper studies image processing technology from the perspective of safe production and operation of coal mines, improves the ability of monitoring and monitoring video through information technology, and ensures the safety of underground mine personnel.

The innovations of this paper are: (1) This paper combines the coal mine underground monitoring with computer vision processing, and introduces the relevant content of image processing in detail. (2) When facing the video in the coal mine, the histogram equalization processing, the dark primary color prior dehazing algorithm and the wavelet transform image enhancement method are used respectively. By evaluating the experimental results, comparing the performance of the three methods, it is concluded that the wavelet transform image enhancement method achieves better enhancement effect.

## Related work

The research and application of computer vision technology came into being with the development of the times. Hamledari H proposes a computer vision-based algorithm that automatically detects components of internal partitions and infers their current state using 2D digital images. The algorithm relies on four integrated shape- and color-based modules, and images are classified into five states based on the results of the four modules. Visual inspection results can potentially provide situational awareness to the construction industry, inform future progress tracking systems about actual status, and help leverage image processing on indoor sites. However, his energy consumption is relatively large^1^. To detect structural damage using the displacements measured in the video, Cha YJ used an unscented Kalman filter to remove noise from the displacement measurements, while detecting damage by identifying the current stiffness and damping coefficient values, given a known mass, used to detect damage. To validate the proposed damage detection method, the state-space formulas are derived without external excitation input and experimentally tested. The experimental results show that the prediction of stiffness and damping characteristics is reasonable and accurate compared with the dynamic analysis calculation. However, he is not based on reality^2^. Garcia CG recommends analyzing pictures through computer vision to detect and analyze people in pictures. Through this analysis, he was able to obtain whether the pictures contained people and to treat the pictures as a sensor with two possible states. As a possible solution, his suggestion is to analyze the entire sequence rather than isolated pictures in order to use pictures as sensors in IoT. However, his accuracy is not enough^3^. Kadir proposes a simplified computer vision-based application that uses an artificial neural network (ANN) that relies on a multilayer perceptron (MLP) to accurately classify wheat kernels as bread or durum. However, his stability is not enough^4^. Nie S reviews the different structures of deep directed generative models and the learning and inference algorithms associated with these structures. His main difficulty in learning and inference using deep directed models with many latent variables is the difficulty in reasoning due to the dependencies between latent variables and the exponential number of latent variable configurations. Quantitative evaluations are performed on benchmark datasets of different models when targeting data representation and feature learning tasks. However, his performance is not high^5^. Khan NA presented a computer vision-based method to identify malaria parasites from light microscopy images. This study addresses the challenges faced in automated detection of malaria parasite tissues. The method he proposed is based on a pixel-based approach, using K-means clustering (an unsupervised method) for segmentation to identify Plasmodium tissues. However, his experimental data is less^6^. Heimberger M He discusses the design and implementation of automatic parking systems from the perspective of computer vision algorithms. He demonstrated that camera systems are critical for addressing a range of automated parking use cases and adding robustness to systems based on active distance measurement sensors such as ultrasonic and radar. The key vision modules that enable parking use cases are 3D reconstruction, parking space marker recognition, free space, and vehicle/pedestrian detection. He details important parking use cases and demonstrates how vision modules can be combined to form a robust parking system. To his knowledge, this is the first detailed discussion of a system view of a commercial automated parking system. However, its scope of application is limited^7^. Ioannidou A surveys methods of applying deep learning on 3D data and provides classifications based on how they are exploited. Based on the results of the inspection work, he concluded that systems that employ 2D views of 3D data generally outperform voxel-based (3D) depth models. However, it can perform better with more layers and severe data augmentation. Therefore, larger-scale datasets and higher resolutions are required. However, his feasibility is not high^8^.

## Image processing method based on computer vision

### Computer vision concepts

Computer vision refers to the use of computers to achieve human visual function, perception, recognition and understanding of the objective world. It includes bionic vision and machine vision. One is to study the mechanism and function of vision by imitating human vision, the other is to study perception and processing, recognition and classification of vision, in order to achieve the purpose of replacing human vision^9^. At present, the visual information receiving equipment mainly includes CCD camera, CMOS camera, X-ray camera, mid-infrared camera, pinhole radar imaging equipment, microwave imager and so on. These devices are connected to a computer to form an optical system.

### Fast image filtering algorithm

The underground environment of coal mines is dark and the imaging of surveillance video images is very poor, which brings a lot of difficulties to the safe production of coal mines, so intelligent video surveillance plays an important role. Due to the poor artificial lighting conditions in the mine, the video image in the mine is not clear and has many limitations. Therefore, before studying the target detection and tracking, it is necessary to enhance the video image. When the image is enhanced, it will be affected by noise, which is likely to amplify the noise and affect the quality of the image. Therefore, many scholars use the wavelet transform technology to deal with the noise problem in the image. The wavelet transform generally adopts the threshold filtering method. The method is simple to operate, and the enhancement effect of the image is also good, but he does not separately process the low-frequency and high-frequency coefficients generated by the wavelet transform, but adopts the same enhancement method. During reconstruction, the enhancement effect is not good, and the edge information of the image becomes blurred. Aiming at these problems, a method based on the combination of wavelet transform and dark primary color prior knowledge is proposed, which can effectively remove image noise while enhancing image detail information, and at the same time solve dense fog, uneven illumination, and improve the image quality after processing. Contrast, highlighting the details of the image, making the enhanced image clearer.

#### Neighborhood pixel smoothing filtering

Due to the randomness of high-frequency noise, the adjacent ratios of noise points and gray-scale pixels show large changes, which can lead to image degradation^10^. Neighborhood pixel smoothing is to replace the gray value of the edit point with the average value of the gray value of the neighborhood of the point to achieve smoothing. Let q(a, b) be the ratio of grayscale pixels in the neighborhood of point (x, y), a = 0,…,c − 1,b = 1,…,c − 1, c is the neighborhood of the number of points of length and width, regardless of whether g(x,y) is the grayscale of the processing point x = 0,…,n − 1,y = 0,…,m − 1, n is the image length, m is the image width, then:

1 $$g(x,y) = \frac{1}{{c^{2} - 1}}\sum\limits_{\begin{subarray}{l} a = 0 \\ b = 1 \end{subarray} }^{c - 1} {q(a,b)}$$

Noise points can be smoothed using formula (1), but at the same time it smoothes the edges of the image, making the edges blurry, something that is not good for edge detection processing. Specifically, the larger the neighborhood, the more blurred it is. Therefore, the following improvements have been made during use.

2 $$g(x,y) = \left\{ {\frac{1}{{c^{2} - 1}}\sum\limits_{\begin{subarray}{l} a = 0 \\ b = 1 \end{subarray} }^{c - 1} {q(a,b)} ,if\left| {\frac{1}{{c^{2} - 1}}\sum\limits_{\begin{subarray}{l} a = 0 \\ b = 1 \end{subarray} }^{c - 1} {q(a,b)} - g(x,y)} \right| > th} \right.$$

The threshold is th. If the difference between the average gray level of the neighborhood and the gray level of the edit point is greater than a threshold, it will be smoothed, if not, the gray level of the edit point will remain unchanged^11^.

This method is simple and requires very little computation, but still produces blur at the edges of the image when used.

#### Fast median filter

The fast median filter algorithm is an improvement on the classic median filter method. It is a nonlinear filtering method, which has a certain ability to keep the limit when filtering, but this ability will be lost as the window increases. When filtering, first select a window, and the window moves from left to right and top to bottom in the picture. For the pixel in the center of each window, all pixels in the window are arranged in grayscale from small to large, and the grayscale is placed in the middle. Since the tail processing takes longer, the processing speed is slower. Fast median filtering does not create a direct queue, but performs histogram statistics on the pixels in the window to determine the median, and only counts columns that move from the left side of the window and columns that move from the right during the shift. Also, statistics are no longer required, increasing processing speed.

#### Fast median filtering method with direction

The above methods are widely used in general image processing and have better filtering effects. However, the neighborhood method damages the edges somewhat during processing^12^. When processing N*M images with a window width of 3 pixels, the computational effort is close to 9 N*M additions and N*M/9 divisions. For fast median filtering, the window is typically at least 3 pixels wide or more for better results. Taking 3 pixels as an example, the calculation amount is equivalent to the calculation amount of the neighborhood method.

Among them, the images are collected in a specific environment F, the quality of each image is relatively consistent, and the filtering work is mainly aimed at the high frequency of the false noise function. At the same time, the requirements for the algorithm are: the important edge information of the image is not destroyed during filtering. The filtered image should be kept clean, without damage to the visual effect, with the characteristics of short processing time and high speed. Based on the above requirements, the fast median filtering method is improved.

**Figure 1:**
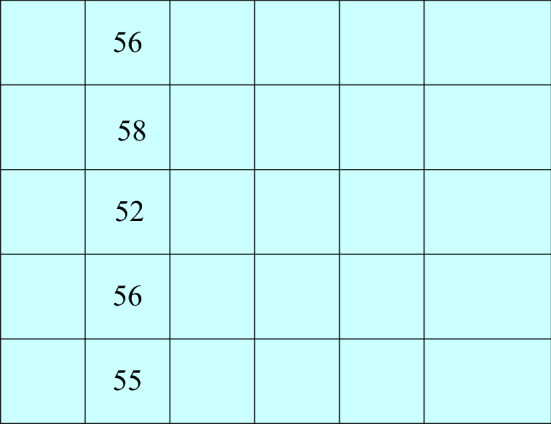
Filter window and grayscale values.

After analyzing a large number of images in the experiment, it is found that the noise points are mainly distributed in the image in the form of discrete points, and the size of the noise points is generally small, mainly one pixel. Therefore, the window of the filter does not need to be very large to reduce the amount of computation using narrow rectangular windows. The selected window size is 1 × 5, as shown in Fig. 1.

### Video image enhancement method in underground coal mine

#### Overview of video image enhancement methods in coal mines

The process of improving the video image of the coal mine involves processing each frame of the video using image enhancement techniques. The processing is mainly divided into two stages: denoising and enhancement. The denoising function is to remove noise interference in the image, and the enhancement function is to improve the clarity and brightness of the video image. It should be noted that when processing video images in coal mines, the image needs to be denoised first and then image enhanced, otherwise, the noise in the image will increase with the improvement of the image.

**Figure 2:**
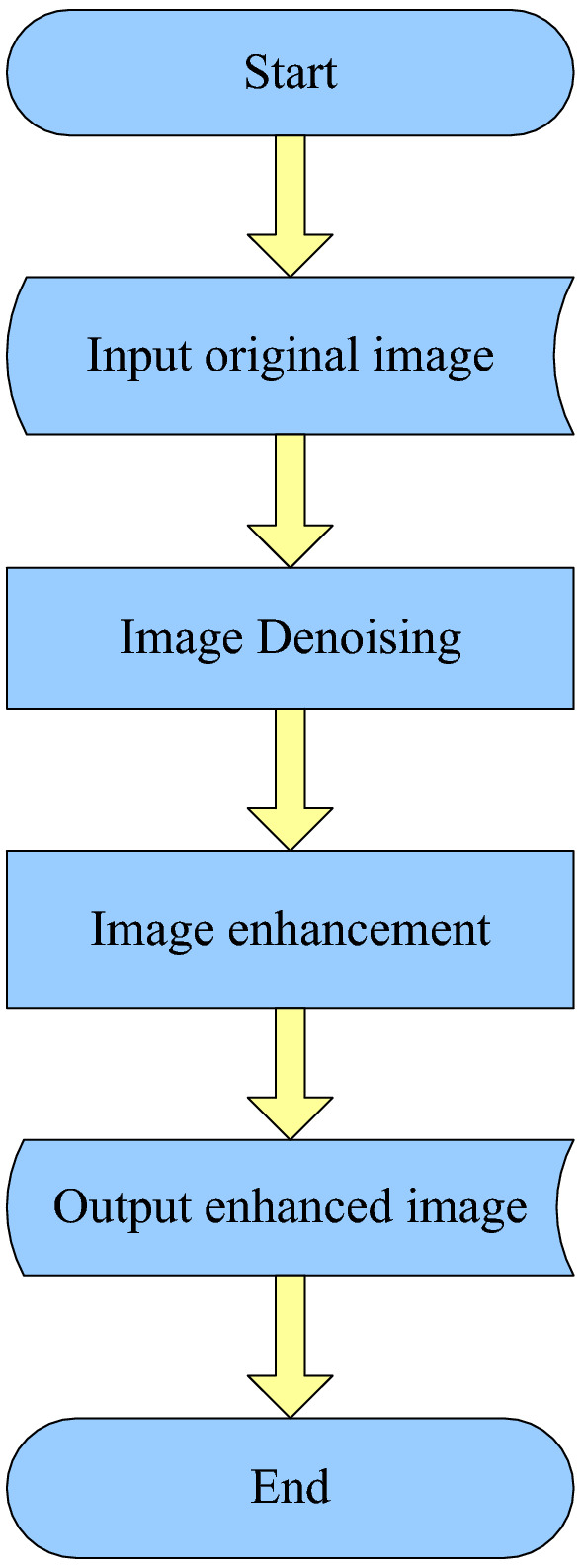
Image enhancement process diagram.

Coal mine video image enhancement is mainly divided into four steps. First, input the video image, then remove the noise in the video image, and then enhance the video image in the mine. Finally, the improved video image is converted into a downhole video image^13^, and the whole operation procedure is shown in Fig. 2.

#### Airspace enhancement method

The air domain, also known as the image domain, refers to the space composed of pixels. Therefore, the spatial enhancement method is actually a convenient and effective algorithm for directly modifying the grayscale of image pixels. Gray level correction algorithm, gray level mapping transformation algorithm, histogram transformation algorithm are its main algorithms. The spatial enhancement algorithm can process the pixels of the whole image, and can also process the sub-images of the image based on the template^14^. Several classical algorithms for airspace will be introduced below.

Grayscale transformation refers to the purpose of stretching the dynamic range of grayscale and improving the contrast by changing the grayscale value of the original image pixels, such as formula (3):

3 $$K(x,y = S[f(x,y)])$$

**Figure 3:**
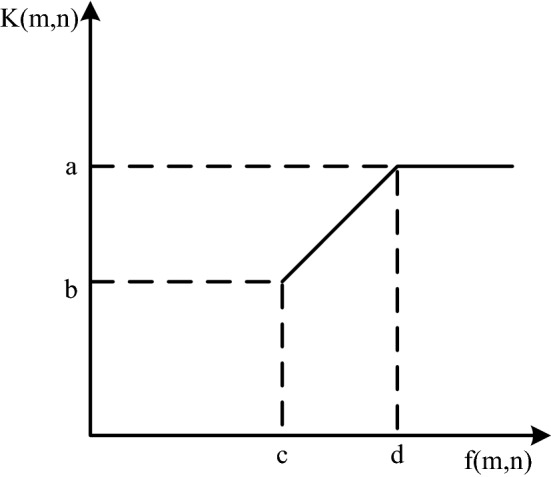
Linearly transformed grayscale image.

In the formula, f (m,n) is the original image, and after the mapping function S, the enhanced image K (m,n) is obtained. The key to this method is the function S. It directly determines whether the grayscale transformation is linear or nonlinear. Linear grayscale transformation, usually applied to underexposure, achieves enhanced effect by linearly extending the grayscale of pixels. As shown in Fig. 3, its expression is as follows:

4 $$K(m,n) = \left\{ {\begin{array}{*{20}l} {b\begin{array}{*{20}c} {} & {} & {} & {0 \le f(m,n) \le c} \\ \end{array} } \\ {\frac{a - b}{{d - c}}[f(x,y) - c] + b\begin{array}{*{20}l} {} & {c \le f(m,n) \le d} \\ \end{array} } \\ {a\begin{array}{*{20}l} {} & {} & {} & {d \le f(m,n) \le L} \\ \end{array} } \\ \end{array} } \right.$$

The function image is as follows:

However, for images with a large dynamic range, linear transformation will lead to the problem of information loss, so nonlinear grayscale transformation is more suitable. At present, the widely used nonlinear transformations are mainly exponential, logarithmic and power transformations^15^. The expressions in turn are:

Exponential transformation:

5 $$K(m,n) = c^{[f(m,n)]}$$

Logarithmic transformation:

6 $$K(m,n) = \log_{c} f(m,n)$$

Power transformation:

7 $$K(m,n) = [f(m,n)]^{n}$$

**Figure 4:**
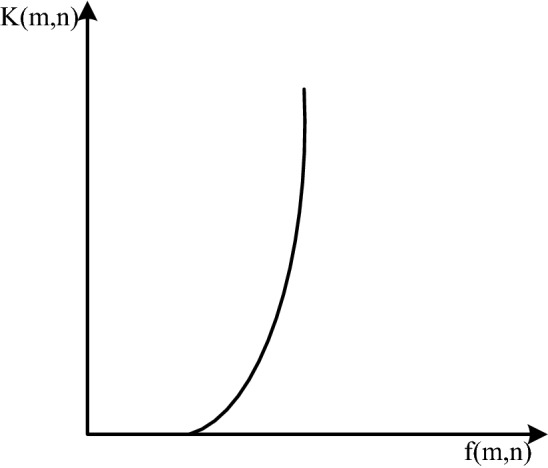
Exponentially transformed image.

**Figure 5:**
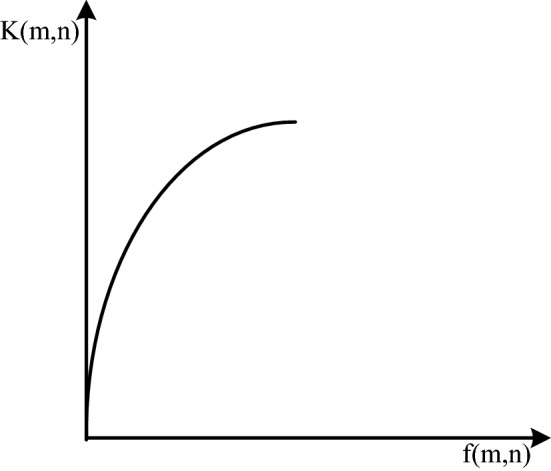
Log-transformed image.

**Figure 6:**
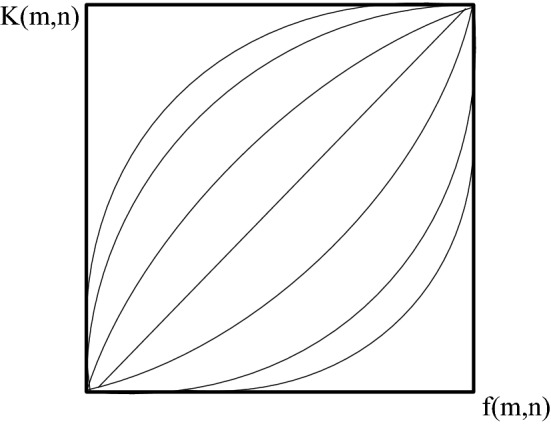
Power-transformed image.

The function images are shown in Fig. 4, Fig. 5, and Fig. 6 in turn:

It can be seen from the function image that the exponential transformation can suppress the low-gray part and enhance the high-gray part. The logarithmic transformation can enhance the low gray part and suppress the high gray part. The power transformation can adjust the γ value to obtain different curves. When γ = 1, it is a linear transformation, and when γ > 1, it is similar to an exponential transformation. The grayscale transformation operation is simple and can quickly adjust the brightness, but the effect of directly applied to the mine image is not good.

#### Histogram equalization

A histogram is a mathematical model used to express an image, which can intuitively reflect the distribution of pixels in an image. The basic idea of equalization is to use normalization to convert the histogram of the original image into a state of uniform distribution, thereby maximizing the dynamic range of grayscale pixel values, thereby improving the overall brightness and contrast of the image. Histogram normalization:

8 $$Q_{T} (T_{j} ) = n_{j} /n\begin{array}{*{20}c} {} & {0 \le } \\ \end{array} T_{j} \le 1,j = 0,1,...,L - 1$$

Cumulative distribution function:

9 $$F = \int_{ - e}^{e} {Q_{T} } (x)dx = \sum\limits_{b = 0}^{j} {Q_{T} } (T_{b} )$$

The relationship between the input image grayscale $v_{b}$ and the output grayscale $T_{b}$:

10 $$T_{b} = P(v_{b} )\begin{array}{*{20}c} {} & {P^{ - 1} (} \\ \end{array} T_{b} ) = v_{b}$$

This function satisfies the previous requirements of the improved function: monotonically increasing, and the probability of the domain and value domain is uniformly distributed between [0, 1]. The relative probability densities are: $Q_{T} (T_{b} )$ and $Q_{v} (v_{b} )$, and the corresponding cumulative distribution functions are:

11 $$CDF_{T} (T_{b} ) = \int_{0}^{{T_{b} }} {Q_{T} (x)dx\begin{array}{*{20}c} {} & {CDF_{b} (v_{b} ) = \int_{0}^{{T_{b} }} {Q_{v} } } \\ \end{array} } (x)dx$$

Functions and distributions of random variables:

12 $$CDF_{T} (T_{b} ) = CDF_{b} (v_{b} )$$

13 $$Q_{T} (T_{b} ) = \frac{{dCDF_{T} (T_{b} )}}{{dT_{b} }} = \frac{{d\int_{0}^{v} {Q_{v} (x)dx} }}{{dT_{b} }} = Q_{T} (v_{b} )\frac{{dv_{b} }}{{dT_{b} }}$$

14 $$\begin{gathered} \frac{{dv_{b} }}{{dT_{b} }} = \frac{{dP(v_{b} )}}{{dv_{b} }} = \frac{{d[\int_{0}^{v} {Q_{v} (x)dx} }}{{dv_{b} }} = Q_{v} (v_{b} ), \hfill \\ Q_{T} (T_{b} ) = Q_{v} (v_{b} )\frac{{dv_{b} }}{{dT_{b} }} = Q_{T} (T_{b} )\frac{1}{{Q_{v} (v_{b} )}} = 1 \hfill \\ \end{gathered}$$

That is, $T_{b}$ means that the density distribution is changed uniformly.

#### Retinex algorithm

The Retinex algorithm is an image enhancement method based on scientific experiments and scientific observations. The principle of Retinex is that the image information obtained by the human eye is not only determined by the absolute light entering the human eye, but the color and brightness around the image also play an important role. At present, the most widely used Retinex algorithms are the SSR algorithm and the MSR algorithm. This theory claims that the images observed by people are mainly affected by illumination and reflection^16^, namely:

15 $$H(x,y) = K(x,y)J(x,y)$$

In the above formula, H (x,y) is the image observed by the human eye, and J (x,y) is the illuminance component, which is affected by the surrounding environment. Z (x,y) is the reflection element and contains the basic properties of the image. The principle of the Retine algorithm is to reduce or even eliminate the influence of the illumination component J (x,y) based on a certain method, and receive the original reflection component Z(x,y) of the image as much as possible. Changed to the logarithmic domain, the reflection component of the object itself is treated as:

16 $$k = h - j = \log (\frac{I(x,y)}{{J(x,y)}})$$

17 $$j = \log (I(x,y) * F(x,y))$$

The principle of the MSR algorithm is to make the calculation of illuminance close to reality through a multi-scale weighting strategy, which can be expressed as:

18 $$\log K(x,y) = \sum\limits_{a = 1}^{A} {\beta_{a} } \left\{ {\log H(x,y) - \log (H(x,y) \oplus G(x,y))} \right\}$$

The number of Gaussian functions is A, and the weight of the nth Gaussian function is $\beta_{a}$, which satisfies $\sum\limits_{a = 1}^{A} {\beta_{a} } = 1$. In general, A is selected as 3, the weights are equal, and the scales are 15, 80, and 250 (large, medium, and small) respectively. Due to the lack of the ability of normalized edge-preserving Gaussian filtering, in recent years, bilateral filtering and guided filtering have been gradually applied to the estimation of illuminance scores, and the results are also ideal in practice.

### Template matching tracking method

The template matching method makes the classic method of target tracking easy. The algorithm has the advantages of simple and easy implementation, wide application range and good anti-noise effect. However, the shortcomings are also obvious, and it is not suitable for scenes with large changes in light and drastic changes in the appearance characteristics of the monitoring target. Importing the video image, use the target template method to find the position of the target to be tracked in the current frame, and the target that matches the template with the highest degree is the target to be tracked. Template matching only needs to compare the template with all sub-regions of the full image. It can determine the region closest to the target, and thus determine the target location. The following will explain the principle of how to realize the comparison between the sub-region and the template. The most direct and convenient method is to calculate the correlation coefficient between the two^17,18^.

Mathematically speaking, (r) is a mathematical distance that describes how close two vectors are. The correlation coefficient uses the law of cosines in mathematics to determine the angle between two vectors, denoted as $\cos (A) = (a^{2} + c^{2} - b^{2} )/2bc$, to calculate the degree of A (the included angle). When r = 1, it indicates that the two vectors are completely similar. When r tends to 0, it indicates that the similarity between the two vectors is lower. When r = 1, the vectors are completely opposite. The cosine theorem is represented by a vector as:

19 $$\cos (A) = < b,c > /(\left| b \right| * \left| c \right|)$$

Which is:

20 $$\cos (A) = (b_{1} c_{1} + b_{2} c_{2} + b_{n} c_{n} )/sqrt[(b_{12} + b_{22} + b_{n2} )(c_{12} + c_{22} + c_{n2} )]$$

The denominator is the modular product of the vectors, and the numerator is the sum of the inner products of the vectors.

In practical use, in order to enhance the correlation of vectors, it is usually necessary to indicate the correlation coefficient^19^. The purpose is to remove the similar parts of the two vectors, specifically the numerator and denominator while removing the mean of each vector, the formula is:

21 $$r = \frac{{\sum {(x_{e} - \overline{x} } )(y_{e} - \overline{y} )}}{{\sqrt {\sum {(x_{e} - \overline{x} } )^{2} (y_{e} - \overline{y} )^{2} } }}$$

Supposing we use the 9*9 target template to match the image, then this last class can be regarded as an 81-dimensional vector, where each dimension represents a pixel gray value in the image. Comparing and matching each word region of the whole picture with the template, and use the correlation coefficient to find the region with the highest degree of matching, so that the position of the target can be determined.

## Experiment and analysis of monitoring system for moving target in underground coal mine

### Improved wavelet transform image enhancement algorithm

**Figure 7:**
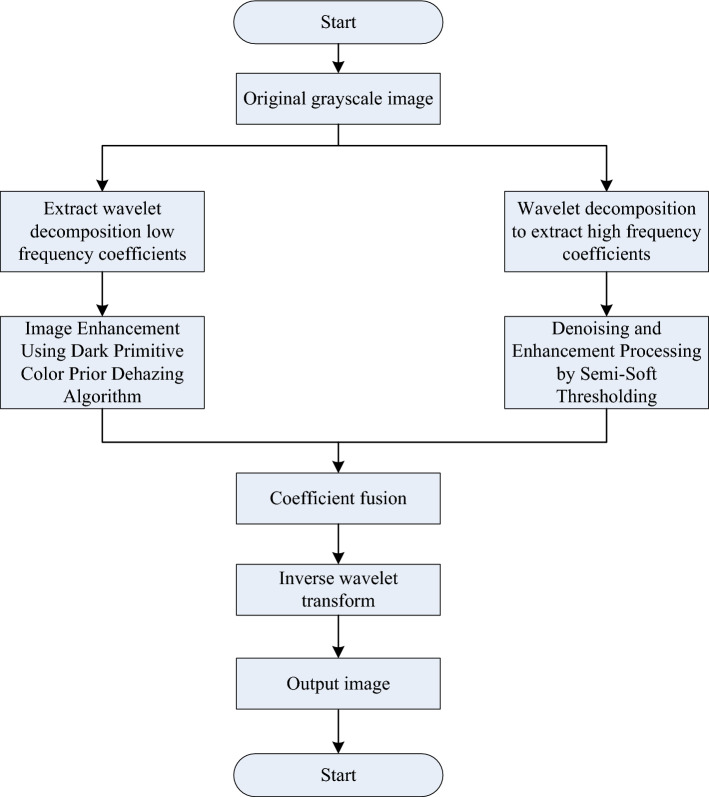
The flowchart of the improved wavelet transform image enhancement algorithm.

This paper mainly enhances the video information in the mine, in order to better achieve the target detection in the coal mine. Aiming at the environmental factors in the mine, this paper proposes an improved waveform enhancement algorithm on the basis of the existing algorithm. Because the image is decomposed by wavelet, the grayscale information that has a great influence on the visual effect mostly exists in the low frequency band, while the noise and details of the image are distributed in the high frequency band. This algorithm uses the dark primary color dehazing algorithm to process the low-frequency coefficients after wavelet transformation, uses the semi-soft threshold filtering method to process the high-frequency coefficients, and finally fuses the two coefficients to reconstruct the enhanced image^20,21^. The improved algorithm steps are as follows:

1. Inputing the low-light image f(x,y);
2. Performing wavelet decomposition on f(x, y) to obtain low-frequency coefficients $c_{1}$;
3. The high-frequency coefficient $c_{2}$ obtained by wavelet decomposition of the low-illumination image f(x, y) is denoised and enhanced by the semi-soft threshold method, and the processed coefficient $c_{3}$ is obtained.
4. Performing dehazing processing on q through the dark primary color prior dehazing algorithm, and obtain the processed coefficient $c_{4}$.
5. Fusing coefficient $c_{4}$ and coefficient $c_{3}$ to reconstruct the image. The flowchart of the improved algorithm is shown in Fig. 7.

### Subjective evaluation

**Figure 8:**
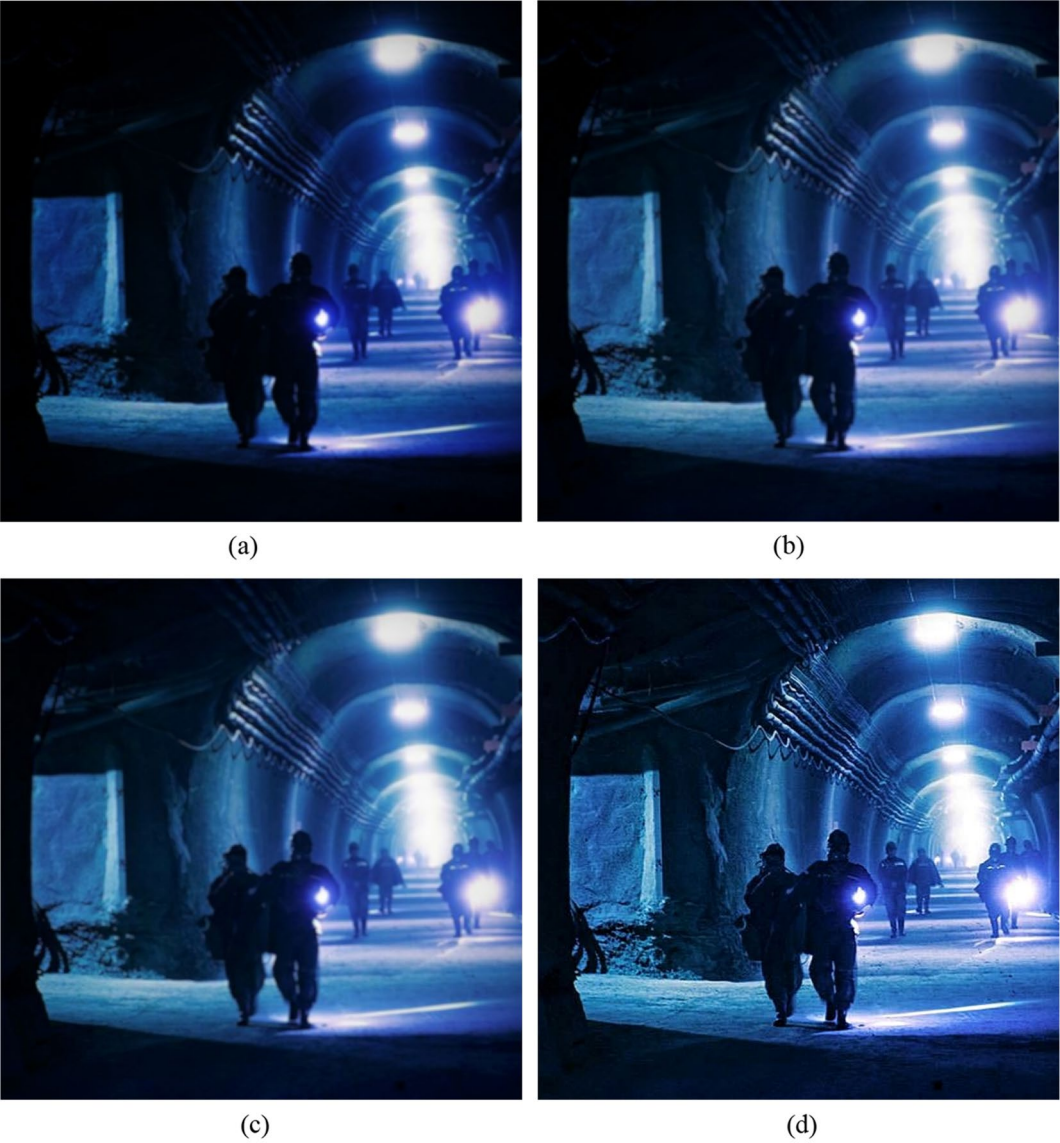
The enhancement effect of the three algorithms on the first set of images in the mine.

Figure 8a–d shows the enhancement results of the three algorithms on the first group of underground images. Figure (a) is the original image without fusion processing. Figure (b) is processed by the histogram equalization enhancement algorithm. Figure (c) is the image processed by the dark primary color prior dehazing algorithm. Figure (d) is the image enhanced by the algorithm in this paper.

From the original image in Fig. 8a, it can be clearly seen that the mine illumination is very insufficient, the video image is not clear, looks gray, and the image quality is not good, and the underground workers and the underground support cannot see clearly. In Fig. 8b of the histogram equalization process, although the brightness is improved, the picture is still unclear. The image processed by the dark primary color prior dehazing algorithm is shown in Fig. 8c. Compared with the original image, the brightness is improved, but the image is distorted and the visual effect is poor. The improved algorithm in this paper is shown in Fig. 8d. The improved algorithm not only improves the brightness of the image, but also the outline of the miner is clearly visible, the shape of the support can also be clearly distinguished, and the quality of the image is improved.

**Figure 9:**
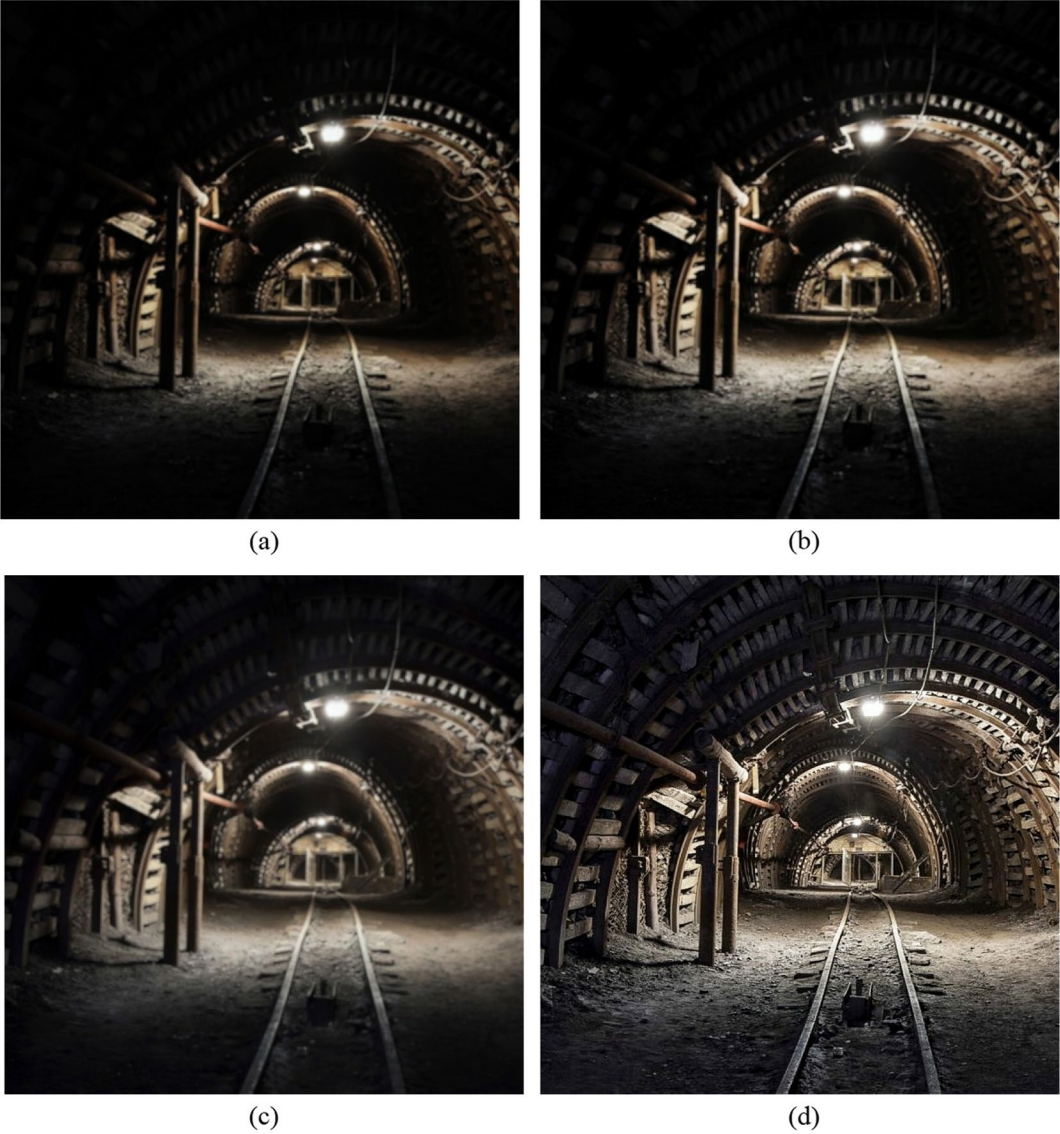
The enhancement effect of the three algorithms on the second set of images in the mine.

Figure 9a–d are the enhancement results of the three algorithms on the second group of underground images.

From the original image in Fig. 9a, it can be seen that the lighting in the mine is dim. Due to the limited monitoring conditions and the influence of dust and coal ash, the video image is very blurred, looks gray, and the quality of the image is not good. From Fig. 9b of the histogram equalization process, it can be seen that even if the brightness of the image is increased, the detail information in the image is still not improved, and the image is still disturbed by dust and dust fog, which does not improve the quality of the image. The image processed by the dark primary color prior dehazing algorithm is shown in Fig. 9c. Compared with the original image, the brightness has been improved and the brightness of the dark area has also been improved. Although the dust and fog in the image are removed, the overall contrast of the image is very low, some edge areas become very blurred, and the edge detail information of the image is obviously lost, resulting in image distortion and poor visual effect. The improved algorithm in this paper is shown in Fig. 9d. The improved algorithm not only improves the brightness of the image, but also clearly sees the outline. The texture and edge of the image are clearer, the details of the image are improved, and the image noise is reduced, so that the quality of the image is improved. The algorithm in this paper can reach the speed of 1.25 s/frame. Compared with the two traditional algorithms, the improved algorithm can achieve better enhancement effect.

### Objective evaluation

The improved algorithm is analyzed from an objective point of view, and two objective parameters are introduced: the information entropy of the image and the average gradient. The information entropy of an image is mainly used to describe the amount of information contained in the image. The higher the information content, the better the image quality. The average gradient is to judge the sharpness of the image. If the gray value in the image changes significantly, the sharpness of the image will be affected. Compared with the traditional enhancement algorithm^22^, the parameters of information entropy and average gradient are shown in Table 1:

**Table 1:** Figure 10 Objective evaluation parameters.

Objective parameter	Information descendant	Average gradient
Original image	5.23	23.45
Histogram equalization	6.25	27.41
Dark primary color a priori dehazing	5.66	22.51
Algorithm in this paper	7.42	35.28

It can be seen from Table 1 that the information entropy of the algorithm in this paper is 31.10% higher than that of the dark primary color prior dehazing algorithm, and 18.72% higher than that of the histogram equalization algorithm. From the average gradient, the algorithm in this paper is 56.60% higher than the histogram equalization algorithm, and 28.71% higher than the dark primary color prior dehazing algorithm.

### Tracking effect of underground workers in coal mines

**Figure 10:**
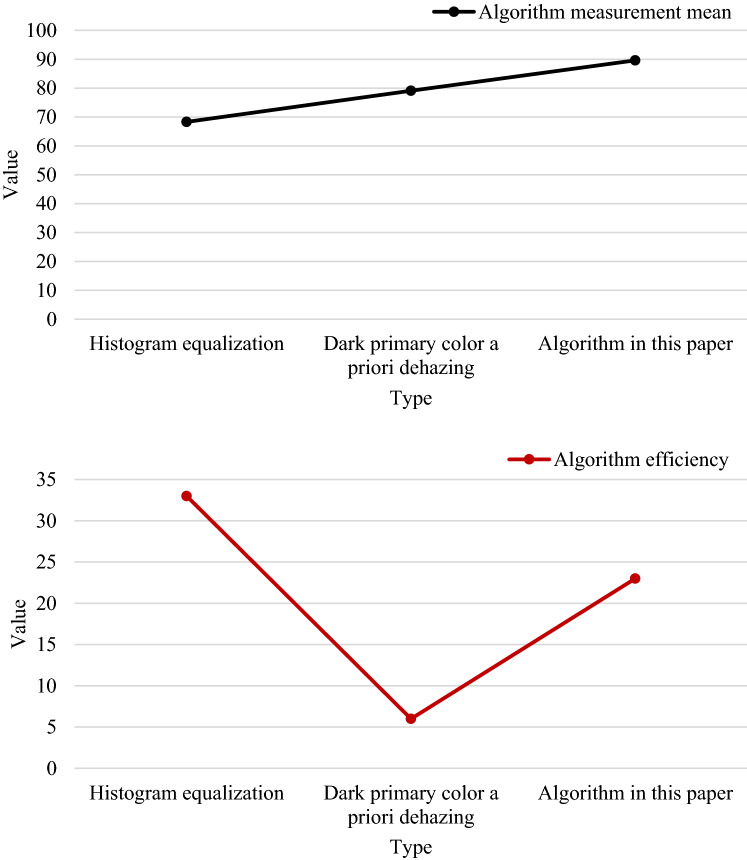
Comparison of the results of three target monitoring.

Generally speaking, the performance of the target monitoring method is based on the similarity between the target area and the actual target area. The superiority of the monitoring method can be judged from two aspects: subjective and objective. Subjective is the direct perception by human vision, and objective is to compare the ratio of the predicted frame of the target with the actual frame^23^. Figure 10 is a comparison of the results of three kinds of target monitoring.

In Fig. 10, the following conclusions can be drawn according to the histogram equalization, the prior dehazing of dark primary colors, and the AP value and FPS of the algorithm in this paper. The dark primary color prior dehazing algorithm has high accuracy, but the running speed is slow and it is not suitable for real-time tracking of targets in coal mines. The histogram equalization algorithm has excellent running speed and accuracy, but under the interference of the complex environment of the mine, the accuracy of the histogram equalization algorithm is greatly reduced to only 68.3%. The wavelet transform image algorithm used in this paper, because the algorithm considers all levels of the detected image, affects the speed of the algorithm to a certain extent, but greatly improves the accuracy, which is ideal for real-time monitoring of coal mine targets.

## Discussion

First of all, through the study of relevant knowledge points of literature works, this paper initially masters the relevant basic knowledge, and analyzes how to conduct research on the monitoring of moving targets in coal mines based on computer vision processing. The concept of computer vision processing and related technical algorithms are expounded, focusing on image enhancement, exploring the method of video image enhancement in coal mines, and analyzing the applicability of image enhancement algorithms in target monitoring in coal mines through experiments.

This paper also focuses on the effect of target monitoring in coal mines. The purpose of the mine moving target monitoring system is to monitor the moving targets in the mine in real time and efficiently, and provide guarantees to provide assistance for coal mine safety production and emergencies. In order to make the designed system meet the advanced nature, integrity and high cost performance of system functions, the requirements of hardware and software should be fully considered^24,25^.

Through experimental analysis, it can be seen that the information entropy of the algorithm in this paper is 31.10% and 18.4% higher than that of the dark primary color prior dehazing algorithm, and 18.72% and 22.44% higher than that of the histogram equalization algorithm. From the average gradient, the algorithm in this paper is 56.60% and 29.33% higher than the histogram equalization algorithm, and 28.71% and 68.26% higher than the dark primary color prior dehazing algorithm. Therefore, the algorithm in this paper can achieve better enhancement effect.

The simulation results show that the enhancement method based on the combination of wavelet transform and dark primary color prior knowledge can enhance the video image in the mine. From the information entropy and average gradient, the improved algorithm is better than the traditional method. Enhanced algorithm; the improved Gaussian background modeling method can realize the detection of people in the mine, reduce the false detection rate, and achieve the purpose of detecting and tracking the moving target in the mine. The above methods have certain advantages for low-light coal mine underground images and personnel positioning. Its theoretical significance provides a realistic basis for the safe production of underground personnel in coal mines.

## Conclusions

With the continuous development of science and technology, intelligent monitoring equipment will be widely used in coal mines and will help ensure the safe operation of coal mines. Due to the shortcomings of traditional mine monitoring equipment, such as the large workload of manual inspection and easy missed inspection, intelligent monitoring equipment is becoming more and more important. Coupled with the complex underground environment of coal mines, where the light is very dim, it is difficult for people to distinguish moving targets, so there will definitely be a regulatory gap. Aiming at the shortcomings of traditional monitoring video processing methods, this paper improves the traditional image enhancement and moving target monitoring methods, and uses wavelet transform and histogram equalization to process video images in coal mines. This effectively monitors the moving targets in the video images of coal mines, and has certain significance for ensuring the safe operation of coal mines, ensuring the safety of underground operations and reducing accidents. However, due to the harsh underground environment of coal mines, only using these three methods to process video images in coal mines cannot fully meet the requirements. How to better deal with video images in coal mines is the main content of future research.

## Data Availability

The data that support the findings of this study are available from the corresponding author upon reasonable request.
